# Autoimmune hemolytic anemia associated with renal urothelial cancer: A case report and literature review

**DOI:** 10.1186/s12894-015-0071-0

**Published:** 2015-07-28

**Authors:** Shuji Isotani, Akira Horiuchi, Masayuki Koja, Takahiro Noguchi, Shouichiro Sugiura, Hirofumi Shimoyama, Yasuhiro Noma, Kousuke Kitamura, Toshiyuki China, Shino Tokiwa, Keisuke Saito, Masaki Kimura, Shin-ichi Hisasue, Hisamitsu Ide, Satoru Muto, Raizo Yamaguchi, Shigeo Horie

**Affiliations:** Department of Urology, Teikyo University, Kaga 2-11-1, Itabashi-ku, Tokyo, Japan; Department of Urology, Juntendo University, Graduate School of Medicine, Tokyo, Japan

**Keywords:** Autoimmune hemolytic anemia, Paraneoplastic syndromes, Renal urothelial cancer, Nephroureterectomy

## Abstract

**Background:**

Autoimmune hemolytic anemia (AIHA) is hemolytic anemia characterized by autoantibodies directed against red blood cells. AIHA can be induced by hematological neoplasms such as malignant lymphoma, but is rarely observed in the urological field. We report a case of renal urothelial cancer inducing Coombs-positive warm AIHA and severe thrombocytopenia that was responsive to nephroureterectomy.

**Case presentation:**

A 52-year-old man presented with a 1-month history of general weakness and dizziness. Hemoglobin level was 4.2 g/dL, and direct and indirect Coombs tests both yielded positive results. Abdominal computed tomography revealed huge left hydronephrosis due to a renal pelvic tumor measuring 4.0 x 4.0 x 3.0 cm, and renal regional lymph-node involvement was also observed and suspected as metastasis. Corticosteroid therapy was administered, and nephroureterectomy was performed. After surgical resection, the hemoglobin level gradually normalized, and direct and indirect Coombs tests yielded negative results. We thus diagnosed warm AIHA associated with renal urothelial cancer.

**Conclusion:**

To the best of our knowledge, this represents the first report of AIHA associated with renal urothelial cancer and severe thrombocytopenia responsive to nephroureterectomy. Renal urothelial cancer needs to be included in the differential diagnoses for warm AIHA, and nephroureterectomy represents a treatment option for AIHA.

## Background

For over 100 years, paraneoplastic syndromes (PNSs) have been recognized as various symptoms associated with certain cancers but not attributable to direct tumor invasion or compression. These conditions have not been well understood until recently. Currently, PNSs are attributed to tumor secretion of functional peptides and hormones or immune cross-reactivity between tumor and normal host tissues [1]. These syndromes may affect diverse organ systems, including the neurological, hematological, endocrinological, dermatological and rheumatological systems. Medical advances have not only improved our understanding of the pathogenesis of PNSs, but have also enhanced the diagnosis and treatment of these disorders. Treatment of the underlying tumor has been reported to often improve such conditions [1, 2].

## Case presentation

A 52-year-old man without any history of gout presented with a 1-month history of dizziness and weakness, and a 3-month history of mild brown gross macrohematuria. Physical examination showed significant pallor of the skin. Microscopic urine examination confirmed red blood cells by directly examining the centrifuged urinary sediment. Hemoglobin level was 4.2 g/dl (normal, 13.0-16.6 g/dl), and mean corpuscular volume (MCV) was 88.2 fl with elevated white blood count (14.7 × 10^9^/l) and normal platelet count (38.0 × 10^9^/l). Absolute reticulocyte counts were 1.1 thou/μL. Prothrombin time and activated partial thromboplastin time were both within normal ranges. The serum haptoglobin level was 15 mg/dL. Lactate dehydrogenase level was elevated to 325 IU/L (normal, 119–229 IU/L). Serum level of total bilirubin was markedly elevated to 4.85 mg/dL (normal, 0.0-1.1 mg/dL), whereas direct bilirubin level was only slightly elevated (0.5 mg/dL; normal, 0.0-0.4 mg/dL). Iron studies showed that B12 level was markedly elevated to 6030 pg/ml (normal, 249–938 pg/ml), whereas folate level was normal. Serum immunoglobulin (Ig) levels were normal, and electrophoresis revealed no abnormal bands.

**Fig. 1:**
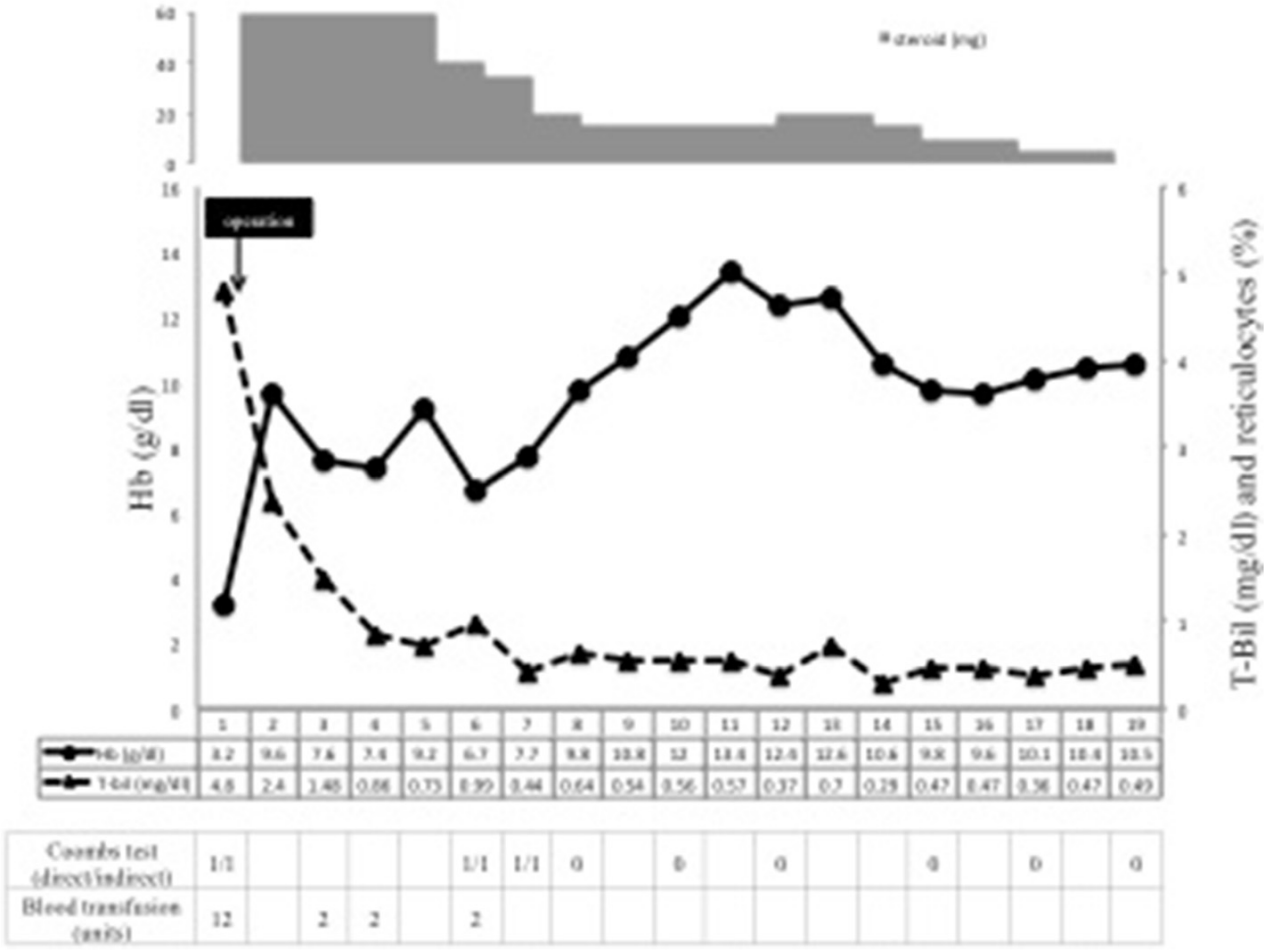
Clinical course of AIHA associated with renal urothelial cancer

A direct Coombs test showed positive results for IgG and negative results for complement C3, while an indirect Coombs test for antiglobulin also yielded positive results. Testing to exclude other autoimmune diseases showed that antinuclear antibodies, antibodies to double-stranded DNA, and antiphospholipid antibodies were all negative. Levels of various tumor markers (prostate-specific antigen, neuron-specific enolase, alpha-fetoprotein, carbohydrate antigen 19–9, carcinoembryonic antigen, squamous cell carcinoma (SCC) antigen were examined and found to be within normal limits. However, serum interleukin (IL)-2 receptor level was elevated to 1503 U/ml (normal, 145–519 U/ml). Urine cytology was negative for cancer. Abdominal computed tomography and magnetic resonance imaging revealed huge left hydronephrosis due to a renal pelvic mass measuring 4.0 × 4.0 × 3.0 cm, and renal regional lymph-node involvement was also observed as a 2-cm mass, which was suspected to represent metastasis of a renal urothelial cancer or malignant lymphoma extension with renal involvement. A diagnosis of IgG-mediated warm autoimmune hemolytic anemia (AIHA) was made on hospital day 3. The patient was transfused with crossmatch-compatible blood. Oral corticosteroid therapy (prednisolone, 60 mg/day) and intravenous Igs 500 mg/day for 3 days were also administered after diagnosis and achieved limited improvement. Surgical treatment was performed on hospital day 7, since tumors that induce AIHA are usually hematological neoplasms such as malignant lymphoma. We planned to eliminate the possibility of malignant lymphoma intraoperatively by intraoperative frozen section diagnosis. If the results of intraoperative biopsy revealed malignant lymphoma of the kidney, initiating chemotherapy as treatment would be considered. Conversely, if some other malignant epithelial tumor was identified, such as renal urothelial cancer or renal cell carcinoma, nephroureterectomy was planned as surgical treatment. The results of intraoperative biopsy indicated reactive hyperplasia of the lymph node, rather than malignant lymphoma. We therefore consecutively performed left nephroureterectomy as a treatment for AIHA. After surgical resection of the left kidney, hemoglobin level and reticulocyte count gradually normalized. Direct and indirect Coombs tests yielded negative results by 7 post-operative weeks. At 10 weeks after surgery, complete blood counts had recovered to within normal ranges and the dose of prednisolone was tapered to 15 mg/day after 10 weeks (Fig. 1).

The diagnosis was confirmed to be warm AIHA associated with renal urothelial cancer. The specimen of the left kidney showed papillary urothelial carcinoma (UC) in the renal pelvis with undifferentiated carcinoma, G2, pT4, INF γ, lt-u0, ew1, ly1, v1, n2), and gradual invasion of the tumor into the renal parenchyma with change to undifferentiated carcinoma. Multiple lung and liver metastases were observed 3 months postoperatively on a CT of the chest and abdomen, so the prednisolone dose was increased to 20 mg/day from 14 weeks to prevent recurrence of AIHA. No symptoms of AIHA or abnormal hemolytic state were observed. Prednisolone was tapered to 5 mg/day and the dosage kept unchanged at 5 mg/day. Although chemotherapy using gemcitabine plus cisplatin for metastases was administered, the patient died 7 months after surgery due to rapid development of metastases and multiple-organ failure (MOF).

## Discussion

Anemia frequently complicates the course of cancer and causes fatigue, representing a very common symptom associated with cancer patients and negatively impacting activities of daily living and quality of life, sometimes becoming life-threating in itself. Some types of anemia associated with cancer involve hematological autoimmune disease, such as AIHA. AIHA is a condition characterized by autoantibodies directed against the red blood cells. Most cases of AIHA are idiopathic, but AIHA can be secondary to some malignancies [3, 4]. These immune-mediated hematological diseases are not usual, but are known as a typical PNS. In the literature, AIHA as a PNS has been demonstrated in patients with a wide variety of tumors, including hematological neoplasms such as malignant lymphoma, squamous cell carcinoma, adenocarcinoma, renal cell carcinoma, oat cell carcinoma and seminoma [4, 5]. However, renal malignancy is rarely associated with hematological PNS, and we have not identified any reports of AIHA associated with renal urothelial cancer.

We examined the literature for AIHA associated with renal tumor, and the results are summarized in Table 1 [1, 3, 5–8]. AIHA secondary to a paraneoplastic process of renal tumor has been reported in association with severe anemia that can lead to significant morbidity and even mortality [7]. From this perspective, correct diagnosis of AIHA as a PNS and start of treatment as soon as possible is very important.

**Table 1:** Contemporary series of AIHA associated with renal tumor

References	Age/Sex	Hb	Stage, Pathology	Cooms test (Direct/Indirect)	Treatment	Response to Treatment	Outcome
Pirofsky B (1968)	65/M	Ht:26%	non-metastatic	positive/positive	steroid	CR	NA
			RCC		nephrectomy		
Spira MA (1979)	68/F	8.7	hypernephroma	positive/positive	steroid	CR	recurrence-free RCC and
				(IgG)	nephrectomy		AIHA for 5 years
					splenectomy		
Bradley G (1981)	57/F	6.8	non-metastatic	positive/positive	steroid	CR	died with lung metastasis after 17 months with AIHA
			clear cell RCC	Non-specific	nephrectomy		
				(only IgG negative)			
Venzano C (1985)	71/F	7.5	lymph-node metastasis,	positive/positive	steroid	CR	NA
			renal cell carcinoma				
Girelli G (1988)	39/M	4.0	T3b	positive/positive	steroid	CR	DAT remained positive, no follow-up data
			clear cell RCC	(IgM)	nephrectomy		
Monga M (1995)	38/F		T1N0M0	negative/positive	nephrectomy	CR	sustained CR of RCC and AIHA for 2+ years
			renal cell cancer				
Lands R (1996)	65/M	8.1	T3a	positive	steroid	CR	NA
			adenocarcinoma		nephrectomy		
Kamra D (2002)	68/F	5.6	T2N0M0	negative	steroid	CR	NA
			clear cell RCC		globulin therapy		
					nephrectomy		
Muñoz-Ibarrav EL (2013)	71/F	6.7	T1aN0M0	positive	steroid		
			clear cell RCC		nephrectomy	CR	NA
Our case (2015)	52/M	3.2	T4N2M0	positive/positive	steroid	CR	died with multiple metastases after 7 months without AIHA
			UC+RCC	(IgG)	globulin therapy		
					nephrectomy		

The diagnosis of paraneoplastic AIHA should be made after ruling out metastases, infections, metabolic processes, and vascular alterations. In this case, we diagnosed paraneoplastic AIHA on day 3 of admission and started treatment, performing surgery on day 7. With that clinical decision, given the disadvantage that this case seems to represent the first report of AIHA with renal urothelial cancer, the literature helped us to determine which treatment might be most suitable, even though the pathogenic mechanisms underlying the association between carcinoma and autoimmune hemolytic disease remain poorly understood. The pathogenesis of these PNSs involves the release of substances with endocrine and metabolic activity by tumor cells, or induction of the release of inflammatory mediators, principally cytokines such as interleukin (IL). IL-6 has been identified as being responsible for some inflammatory processes [4]. In this case, such mediators were suggested to be secreted from cancer cells based on blood analysis finding abnormal elevations of the white blood count, B12 level, and serum IL-2 receptor level on admission. These findings also supported our diagnosis of AIHA as a PNS in this case.

Steroids represent the major therapy for idiopathic AIHA. Steroid-resistant cases may require splenectomy, although more recently other immunosuppressants such as azathioprine, mycophenolate, the monoclonal antibody rituximab, and intravenous immunoglobulins have been successfully applied. Control of the carcinoma through tumor excision or chemotherapy with corticosteroid therapy and/or splenectomy might improve hematological abnormalities [8]. In fact, most reports with renal tumor seem to have described nephrectomy as an effective treatment for AIHA and renal carcinoma [1, 3, 5–8].

In our case, we performed nephroureterectomy to control the AIHA rather than the cancer. Surgical treatment improved the hemolytic state for at least 7 months, even though cancer control failed, with multiple metastases appearing in the liver and lungs within 3 months. We considered increasing the prednisolone dose from 15 mg/day to 20 mg/day to prevent the recurrence of AIHA, because we thought that recurrence of the carcinoma might initiate the hematological abnormalities. If the tumor started to progress, AIHA would thus presumably recur. However, no AIHA symptoms were observed, with negative Coombs reactions observed until the patient died of MOF. This result suggests that once control of AIHA was initiated by surgical intervention, control could be maintained with steroid therapy alone.

## Conclusions

We have reported a case of warm AIHA as a PNS with renal urothelial cancer. Hemolytic anemia as a PNS is diagnosed by excluding other causes such as primary hematological alterations, metastasis, and vascular processes. The appearance of unusual hematological alterations in patients with renal tumor should be suspected with these hematological paraneoplastic alterations. Our experience suggests that hemolytic anemia as a PNS should be considered in patients with hematological alterations with renal urothelial cancer, and if confirmed, surgical intervention with steroid administration should be considered.

## Consent

Written informed consent was obtained from the patient for publication of this case report and the accompanying images. A copy of the written consent is available for review by the editor of this journal.

## Abbreviations

AIHA: Autoimmune hemolytic anemia
PNS: Paraneoplastic syndromes
MOF: Multiple-organ failure
UC: Urothelial carcinoma
IL: Interleukin
